# Formal finance and household enterprise performance in Ghana: The gender dimension

**DOI:** 10.3389/fpsyg.2022.887545

**Published:** 2022-09-30

**Authors:** Yiqing Peng, Charles Komla Delali Adjasi

**Affiliations:** ^1^Department of Economics, Econometrics and Finance, Faculty of Economics and Business, University of Groningen, Groningen, Netherlands; ^2^University of Stellenbosch Business School, University of Stellenbosch, Stellenbosch, South Africa

**Keywords:** women, entrepreneur, formal finance, household enterprises, productivity

## Abstract

In this study, we empirically examine the gender dimension of the effect of formal finance on enterprise performance. While the literature suggests that formal finance, in general, improves firm performance, this effect may differ across firms headed by male and female entrepreneurs since the latter are faced with more severe social, economic, and financial constraints, which undermine their firm performance. Consequently, the effect of finance on firm performance is expected to be weaker in female-headed enterprises. So far, there is little evidence as to whether a gender gap exists regarding the effect of formal finance on firm performance, especially among small household enterprises. To fill the gap in the literature, we use the Ghana Living Standards Survey 2016/2017 and study the effect of formal credit on the productivity of male-headed and female-headed non-farm household enterprises in Ghana. Our results show that a positive effect of formal credit on labor productivity is only found in male-headed enterprises, but not in female-headed ones. We suspect that this result may be explained by women’s relatively less endowment in conditional factors, such as skills, knowledge, experiences, and capabilities.

## Introduction

Gender equality, in general, refers to equal rights, responsibilities and opportunities of women and men. Since it is often more difficult for women to access and use resources needed for their personal development, e.g., education, health, and employment, gender inequality remains an issue to be addressed globally. That is also why in 2015 the United Nations adopted achieving gender equality and empowering women and girls as one of the Sustainable Development Goals (SDGs).^1^

In this study, we use the Ghana Living Standards Survey Round 7 (henceforth GLSS 7) and investigate whether using formal finance helps female heads of non-farm household enterprises to achieve better firm performance. Our findings are twofold. First, non-farm household enterprises, which applied for and finally managed to obtain credit from banks and other financial institutions, have higher productivity than those that did not apply for credit. Second, there is a gender gap with respect to the effect of formal credit on firm productivity. Formal credit exerts a positive effect on productivity only in male-headed enterprises, but not female-headed ones.

We contribute to the literature in three aspects. First, while the literature highlights the heterogeneity between male and female entrepreneurs in terms of business knowledge, skills, and experience, as well as personality traits, such as entrepreneurship and attitude toward risk (Bardasi et al., 2011), most empirical work does not examine the gender dimension. In this study, we explicitly look at the role of gender. We suspect that entrepreneurs need to have certain knowledge, skills, and personality traits in order to use credit productively and reap the benefits. Since female entrepreneurs tend to have less resources, technology, and education compared with male entrepreneurs, a lack of these complementary factors means that the effect of credit on firm performance will be limited among female-headed enterprises.

Second, different from the empirical studies that investigate the effect of finance on firm performance of small and medium-sized enterprises (see, e.g., Fowowe, 2017; Bokpin et al., 2018), we focus on microenterprises, which are often not registered by authorities formally, and whose business activities are largely domiciled in households. In fact, microenterprises in the informal sector play a critical role in developing countries. According to International Labor Organization, employment in the informal sector takes up 85.8% of total employment in Africa and makes a major contribution to national GDP.^2^ However, not much is known how formal finance imposes an effect on the performance of microenterprises in the informal sector in African countries. We aim to fill this gap in the literature.

Third, this study is related to a small strand of the literature that examines the effect of finance on firm performance in the Ghanaian context. To illustrate, Osei-Assibey (2013) investigates whether sources of finance, that is, whether internal finance from savings and retained earnings or external finance from formal/ semi-formal financial institutions have a different effect on firm productivity. Fafchamps et al. (2014) study how cash and in-kind grants impact the profit of microenterprises in urban Ghana using a randomized controlled trial. We complement Fafchamps et al. (2014) by focusing on the use of formal credit, i.e., credit from banks and other formal financial institutions, which is different from cash or in-kind grants. The latter does not require repayment, which leads to a weaker incentive for enterprise to improve firm performance compared with loan financing. We are interested to understand how formal finance, if it can be used by household enterprises, will influence firm performance. This question is particularly relevant for Ghana because formal financial institutions are where household enterprises have the most difficulty in getting financed.

The remainder of this chapter proceeds as follows. In Section “Literature review” we review the relevant literature. In Section “The Ghanaian context”, we provide a description of microenterprises and access to finance in Ghana. In Section “Methodology and data”, we discuss the methodology adopted in our empirical analysis and provide a description of our data set. Section “Results” discusses the estimation results. The paper concludes in Section “Conclusion”.

## Literature review

Finance is important for firms. A lack of finance means that establishing an enterprise is difficult in the first place. Despite promising business ideas, entrepreneurs are not able to get sufficient funds from the financial sector to carry them out. Financial constraints also negatively influence the existing enterprises. Credit inaccessibility hinders entrepreneurs from investing in new production facilities, e.g., machinery, equipment, and technology, and hiring new employees to expand business operation and production, which hampers firm growth. In fact, many enterprises claim finance as their main obstacle to firm growth (see, e.g., Beck and Demirgüç-Kunt, 2006; Ayyagari et al., 2008; Kuntchev et al., 2013).^3^ Empirically, Beck et al. (2005) find that financing barriers impose an adverse effect on firms’ sales, especially for small-sized enterprises.^4^ Beck et al. (2006) shows that firm size, indicated by total sales, is positively associated with the amount of credit issued to the private sector by financial intermediaries. Fowowe (2017) finds that financial constraints hinder firms’ employment of permanent workers in 30 African countries.

Productivity is another critical dimension of firm growth. As the literature on finance and growth (see, e.g., King and Levine, 1993; Levine, 1997, 2005) suggests, the mechanism by which finance contributes to economic growth is through productivity enhancement. Hence, the issue of alleviating firms’ financial constraints has received much attention (see, e.g., OECD, 2006). Empirical evidence has also established a positive link between finance and productivity. At the macro-level, the literature looks at country-level data and examines the effect of finance on aggregate productivity. For example, Beck et al. (2000) and Rioja and Valev (2004) show that credit expansion to the private sector is positively related to total factor productivity (henceforth TFP) growth in the economy. At the micro-level, the literature uses firm-level data to study the relationship between access to finance and firm productivity.^5^ Anos-Casero and Udomsaph (2009) show that firms’ TFP growth in eight Eastern European countries is positively related to access to finance, that is, the degree to which firms are able to get financed from the formal sector (both domestic and foreign banks), and the informal sector, such as family or friends.^6^ Bokpin et al. (2018) investigate 15 Sub-Saharan African countries and reveal that having access to a credit line or an overdraft facility improves firms’ TFP growth. Ferrando and Ruggieri (2018) examine 7 euro-area countries and find that financially constrained firms tend to have lower labor productivity. Levine and Warusawitharana (2021) look at France, Italy, and Spain and show that firms’ financial frictions, with respect to leverage, cash holdings, and interest expense, tend to drag firms’ TFP growth. Gatti and Love (2008) use a dataset of 548 firms in Bulgaria and find that firms’ TFP is positively associated with the degree of financial access. Similarly, Galasso et al. (2018) show that financial constraints drag the productivity of firms in the manufacturing industry in Italy. Krishnan et al. (2015) use banking sector deregulation in 1990 in the United States as a natural experiment and find that increased access to bank financing raises firms’ TFP. This effect is more significant for financially constrained firms.

While access to finance is found to improve firm performance in general, this effect may differ across firms headed by male entrepreneurs and female entrepreneurs since the latter are faced with more severe social and economic constraints, which undermine their firm performance. Carranza et al. (2018) conduct a comprehensive literature review of the constraints that explain the heterogeneity in business performance between male and female entrepreneurs. Below, we provide a summary of their findings.

To start with, male entrepreneurs and female entrepreneurs are different with respect to the motivation to set up their business, goal of firm growth, attitude toward risk, and personality traits. First, women are more likely to enter self-employment due to economic necessity, e.g., low household income or unsatisfactory earnings from wage employment, rather than a creative business idea. Second, women usually have a lower expectation with respect to the growth of their business, as they prefer a work-life balance over stress and pressure that they must overcome to meet their business outcomes. Third, women are often more risk-averse than men. Consequently, women are more likely to stay away from high risk–return projects, even though risk is an unavoidable part of any business activity. Fourth, women are less competitive. Compared with men, they emphasize more on achieving a sense of fulfillment, rather than financial profitability, independence, and position.

Next, male entrepreneurs and female entrepreneurs are endowed differently with respect to assets, knowledge and business skills, and social networks. First, women, in general, have less assets, such as land, property and productive assets (e.g., cattle or goats), as they have fewer years of employment due to pregnancy, childbirth and household care, suffer from wage discrimination in the labor market, or are even forbidden to hold assets by law in some countries. As a result, female entrepreneurs are less likely to access formal credit and expand their business due to a lack of collateral. Second, women usually have less education, management skills, and business experiences. Since entrepreneurs need to acquire a certain level of knowledge and skills first before they can identify prospective business opportunities, adopt innovations, and implement business ideas, a lack of these essentials means that female entrepreneurs have to operate in less profitable sectors where a lower level of human capital is required. Third, the social networks of female entrepreneurs are often composed of relatives and friends, who are also females. A lack of connections makes it difficult for them to gain information and enter more profitable sectors, such as science, technology, manufacturing, or other high-order services.

In addition, women are faced with external constraints arising from the legal system, social norms and culture, discrimination from financial institutions, and the responsibility of taking care of family. First, legal institutions sometimes put women at a disadvantaged position.^7^ For example, women are not allowed, by law, to open bank accounts without the permission of their husbands in Nigeria, Chad, and Guinea-Bissau. Women in some African countries are deprived of their right to possess property, e.g., by inheritance, or manage property to finance their businesses. Obviously, discrimination against women by the legal system discourages female entrepreneurship and adversely affects their firm growth. Second, social norms may also be discriminatory against female entrepreneurs. For example, in many cultures it is unacceptable for women to take control of finances, to have contact with men outside of their household, or to spend time and effort in running their business, which is often viewed as a man’s responsibility. Undoubtedly, such a social environment restricts the growth potential of female-headed enterprises. Third, women are more likely to be discriminated by financial institutions, which can be seen by the fact that female entrepreneurs are required to have more collateral or pay higher interest rates to secure a bank loan. Fourth, women usually take up the responsibility of taking care of their family, which limits the time and efforts they can devote to entrepreneurial activities, leading to lower profitability of female-headed enterprises. Besides, evidence shows that some female entrepreneurs tend to use bank loan for household expenditures, rather than invest in their business as supposed to be, due to the pressure of fulfilling domestic duties. This way of using credit by women limits the effect of finance on firm performance.

Hence, we expect that the effect of finance on firm performance is weaker in female-headed enterprises. The idea is that while finance allows female entrepreneurs to invest in new facilities to enhance the efficiency in production and firm performance, this effect will be weakened by the fact that female entrepreneurs are bounded by certain social and economic constraints as we have summarized them above. A lack of business ambition, confidence, tolerance of risk, business knowledge and skills, social networks, protection by the legal system, and support from family or community makes it difficult for female entrepreneurs to make the best use of finance and reap the full benefits.

Recent evidence from field experiments seems to support our expectation. For example, De Mel et al. (2008) conduct a randomized experiment in Sri Lanka, where they provide cash grants to microenterprise owners and investigate the impact of access to cash investments on the profitability of the microenterprise. They find that grants create large profit increases for male entrepreneurs, but no increase in profit is found for female entrepreneurs, even though women are often believed to be more credit constrained than men in low-income countries. Fafchamps et al. (2014) conduct a field experiment, where they provide cash and in-kind grants to the owner of microenterprises in Ghana. For female-led microenterprises running at the subsistence level, they do not find a gain in profits from getting either cash or in-kind grants. For larger female-led microenterprises, only in-kind grants exert a positive impact on profit growth, while cash grants do not. The main difference between our study and Fafchamps et al. (2014) is that we focus on the use of formal finance, i.e., credit from banks and other formal financial institutions. Cash or in-kind grants and formal credit are different. Cash or in-kind grants financing does not require repayment from the enterprises, while loan financing requires repayment with interest. Hence, they exert different incentives for enterprises to improve firm performance. We are interested to understand whether formal finance, if can be used by household enterprises, has a positive effect on firm performance. This is particularly relevant for Ghana, because formal financial institutions are where household enterprises have the most difficulty in getting financed.

Based on the above discussion, we derive the following hypotheses:

*H1:* Household enterprises, which applied for and finally obtained credit from banks or other financial institutions, have higher productivity.

*H2:* The positive effect of obtaining formal credit on productivity is weaker for female-headed household enterprises.

## The Ghanaian context

An important type of business in Ghana is microenterprises, in which no more than four people are hired (Mensah et al., 2007; Masakure et al., 2008).^8^ Typically, these microenterprises specialize in small-scale production and operate in the informal sector, where paid production and sale of goods and services are legitimate in all respects besides the fact that the businesses are unregistered by or hidden from the state for tax and/or benefit purposes (Williams and Nadin, 2010). A particular form of microenterprises is household enterprises, whose activities are domiciled in households, and which are usually unregistered.

To illustrate, the most recent survey on households in Ghana, i.e., The Ghana Living Standards Survey 2016/2017 (GLSS7) indicates that 43.5% of Ghanaian households own or operate a non-farm household enterprise. Despite a drop from 51% of households in 1992, the proportion of households engaged in non-farm enterprises and the growth in non-farm enterprises over the years show the importance of these enterprises to the economic development of the country. Indeed, at an estimated number of 3.825 million enterprises, and with a household population of 28.4 million in 2017, the level of non-farm household enterprise activity is very dense. These non-farm household enterprises operate mostly (close to 90% or more) without formal documentation.

A critical challenge faced by the microenterprises in Ghana is access to finance from commercial or rural community banks (Nkuah et al., 2013). Particularly, credit constraint is severe for non-farm household enterprises (Ackah, 2013). The GLSS 7 reports that 56% of non-farm household enterprises consider access to credit as the most challenging factor when doing business. Household savings form a major component of capital for most of these enterprises (69% in the 2017 survey). An average of 60% of non-farm household enterprises have cited access to credit as a major challenge in most of the surveys. Obviously, Ghana’s financial system has not enhanced access to credit in a way that can be of utmost benefit to such non-farm household enterprises.

Consequently, firms have to consider other sources of finance (Osei-Assibey et al., 2012). First, firms can make use of internal finance, i.e., firm’s savings and retained earnings, which, in general, incurs no transaction costs and does not require disclosing firms’ information. Alternatively, firms can turn to external finance from semi-formal financial institutions, i.e., non-bank institutions, such as NGOs, savings and loans companies, credit unions and cooperatives, government agencies, and microfinance institutions. Compared with banks, semi-formal institutions are more willing to provide loans, lend to women and the poor, and reduce collateral requirements, thus particularly catering to firms that are unable to get financed formally. Lastly, microenterprises can rely on informal finance from institutions that are not regulated by Ghanaian banking laws, such as money lenders, SUSU or ROSCA operators, friends, and relatives, or from other resources, also known as bootstrap financing. One important example of the latter is trade credit.

Nevertheless, Osei-Assibey (2013) highlights that bank finance in Ghana has several distinct advantages. First, banks are able to provide long-term finance that allow firms to invest in new capital and modern technologies to promote production without relying on working capital. Long-term finance is usually not available from other sources. Second, banks are specialized in monitoring firm investments and exerting corporate governance, which help microenterprises to be organized, managed, and operated in a productive manner. Third, the interest-bearing nature of bank finance incentivizes firms to make an extra effort to develop. In contrast, the favorable lending conditions offered by informal finance, e.g., interest-subsidized loans or free grants, make the effect of credit on firm performance less prominent.

## Methodology and data

### Data and sample

To conduct empirical analysis, we draw data from the Ghana Living Standard Survey 2016/2017 (GLSS7) conducted by the Ghana Statistical Service, which is a nation-wide survey that provides us with comprehensive information about households in Ghana, including demographic characteristics, education, health, migration, and finance.^9^ We focus on non-farm household enterprises, which is reported in Section 10 of GLSS 7. Specifically, Section 10 provides information on the characteristics of non-farm household enterprises, such as how long an enterprise has been operated, how much output it has produced, how much revenue it has generated, whether it is formally registered, and whether it hires household members and apprentices. Besides, this section provides information about the “finance” of household enterprises, including the main source of capital to establish an enterprise, the nature of the capital, and whether the entrepreneur get credit from formal financial institutions. This allows us to investigate the relationship between us of formal finance and firm performance, which is the core of the present study.

**Figure 1:**
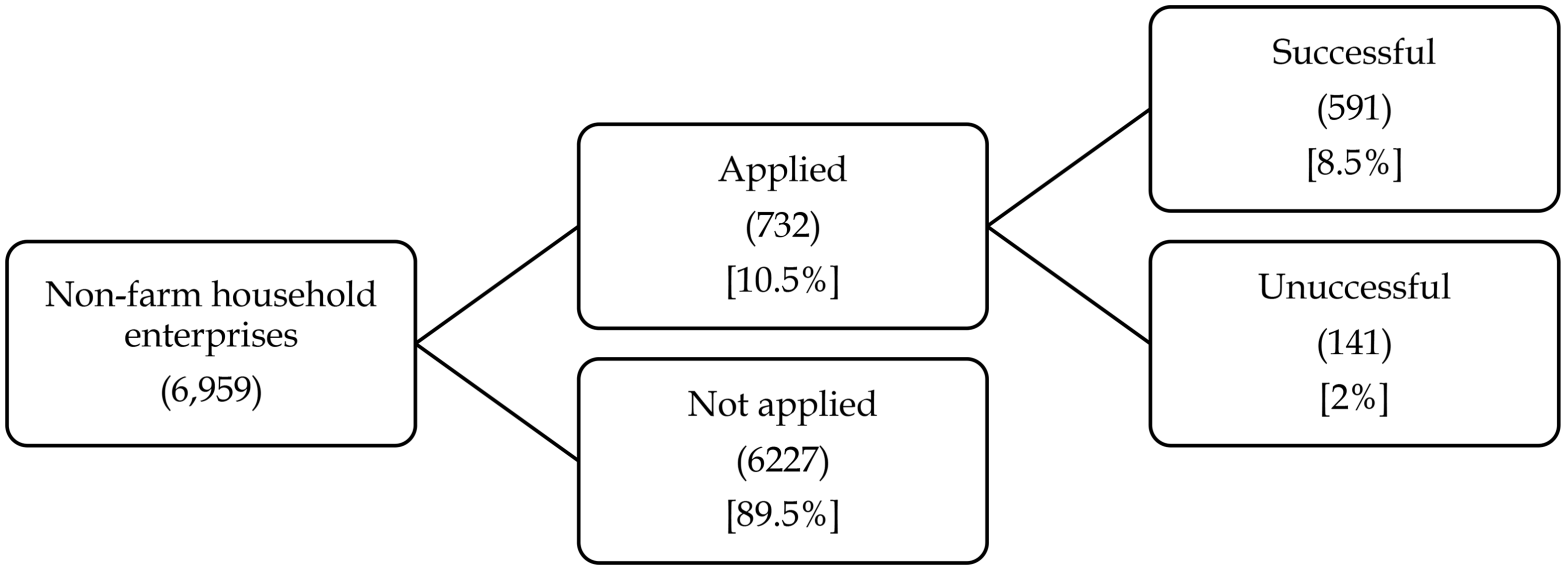
Use of credit from banks and other financial institutions.

**Figure 2:**
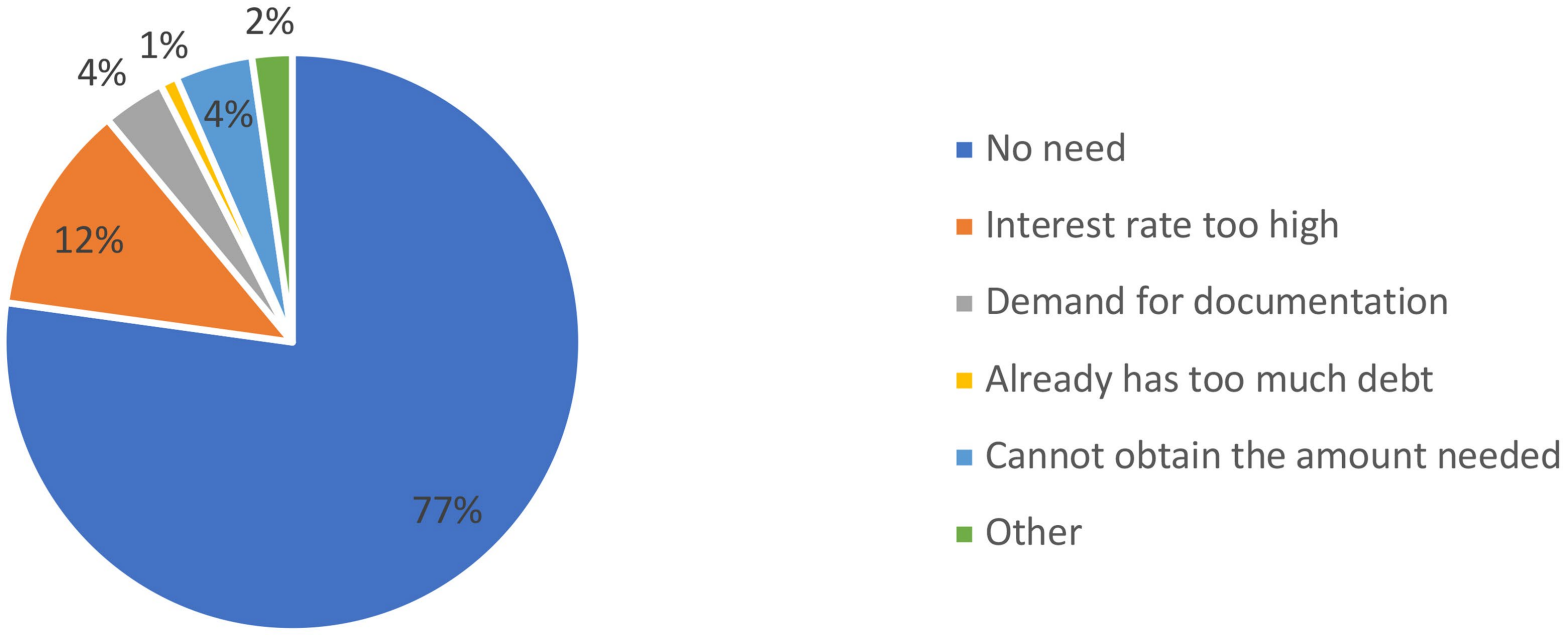
Reasons for not trying to obtain a loan.

Our sample includes 6,959 non-farm household enterprises, among which 97.5% of the enterprises hired <5 workers. Only about 1.8% of the household enterprises hired 6–10 employees. On average, household enterprises hired 1.67 employees, which confirms the predominance of microenterprises in Ghana. The median size of a household was 3 members. About 63% of the household heads were male (with an average age of 55) and 37% are female (with an average age of 50). When it comes to financial usage, 89.5% of non-farm household enterprises did not even apply for formal credit (see Figure 1). Figure 2 presents the reasons why household enterprises did not apply for credit. The main reason is that they did not need credit (77%). The second main reason is that the interest rate was too high (12%). The third main reasons are that they did not meet the requirement of documentation (4%) and that they could not obtain the amount of credit they need (4%), followed by that they had already had too much debt (1%) or other reasons (2%). Among enterprises that did apply for formal credit, about 80% of the enterprises succeeded and 20% of them failed. Overall, only 8.5% of non-farm enterprises in our sample used formal credit.

### Empirical strategy

To test our hypothesis, we adopt the following econometric model:


(1) $${\mathrm{Productivity}}_{i}=\beta_{0}+\gamma \mathrm{Financ}e_{i}+\theta X_{i}+\varepsilon_{i}$$

where *i* refers to household enterprise and *ε* is the error term.

#### Dependent variable

The dependent variable is Productivity, which is measured by the labor productivity of household enterprises. It is calculated as the total value of sales divided by the number of workers hired.^10^ We focus on productivity, rather than the other dimensions of firm performance, e.g., firm size, sales, or profitability, because lagging productivity growth has been claimed as the main constraint for economic development in Africa (see, e.g., Wolf, 2007; Naanwaab and Yeboah, 2013; Wamboye and Tochkov, 2015). We are interested in whether formal finance helps non-farm household enterprises to improve the efficiency of their production.

#### Independent variables

The key explanatory variables are captured by the vector **Finance**, which include three dummy variables that indicate an enterprise’s use of formal finance based on the response to the question “During the past 12 months, has this enterprise tried to get credit from banks, and other financial institutions?.”^11^ Specifically, these dummy variables are (1) Notapplied, which equals to 1, if the enterprise did not apply for formal credit, otherwise 0; (2) Successful, which equals to 1, if the household enterprise applied for formal credit and succeeded, otherwise 0; and (3) Unsuccessful, which equals to 1, if the household enterprise applied for formal credit, but failed, otherwise 0.

In the baseline analysis, we treat household enterprises that did not apply for credit, namely Notapplied = 1, as our reference group. We are interested in the coefficient for the variable Successful, which is expected to have a positive sign indicating that, compared with household enterprises that did not apply for formal credit, household enterprises that applied for and managed to get credit are expected to have a higher level of labor productivity (Hypothesis 1). To test the gender effect (Hypothesis 2), we further conduct a subsample analysis by splitting our sample into male-headed group and female-headed group and repeating the previous analysis in each group. The coefficient for the variable Successful is expected to differ in magnitude and/or statistical significance between the male and female subsamples.

One issue with the baseline analysis is that heterogeneity exists between enterprises that managed to obtain credit, i.e., the group of interest, and enterprises that voluntarily excluded them from obtaining credit, i.e., our reference group, because the latter might know they would not be able to get credit even if they applied, or they simply might not need credit. Considering the selection bias present in the reference group, we conduct robustness checks by repeating our baseline analysis based on a small sample that consists of enterprises that applied for credit only. We treat household enterprises that applied for but failed to obtain credit, namely Unsuccessful = 1, as our reference group. Again, we are interested in the coefficient for the variable Successful, which is expected to be positive indicating that, compared with household enterprises that applied for credit but failed, household enterprises that applied for and managed to get credit are expected to have a higher level of labor productivity.

**X** is a vector of control variables, which are suggested by recent empirical studies that aim to explain firm productivity (see, e.g., Gatti and Love, 2008; Ayyagari et al., 2010; Du and Girma, 2012; Osei-Assibey, 2013; Fowowe, 2017; Adegboye and Iweriebor, 2018; Bokpin et al., 2018). To start with, we control for household head characteristics, which include age (Age), gender (Male), education (Schooled), and marital status (Married). First, business experiences are expected to improve as household heads become senior. Thus, we expect a positive link between the age of household heads and their firm performance. Second, as noted in Section “Literature review”, female entrepreneurs are faced with more social and economic constraints that tend to drag their firm performance in general. Thus, we expect that firm productivity is lower for female-headed enterprises. According to GLSS 7, the head of household is identified by the household members themselves. He or she is the person who is named in reply to the question ‘Who is the head of this household?’ Most often, but not always, it will be the person who is the main provider and who is familiar with all the activities and occupations of household members. The head of household can be male or female. Third, household heads, who have attended school, tend to have more training and, thus, are more likely to use credit productively compared with unschooled counterparts. Hence, we expect that firm performance is better in enterprises whose household heads have attended school. Fourth, household heads, who are married, are expected to bear additional responsibility for taking care of their family and have less time and effort that can be otherwise devoted to their enterprise. Hence, productivity is expected to be higher in enterprises whose household heads are unmarried.

Next, we control for enterprise characteristics, including firm age, i.e., the number of years that the household enterprise has been operating (Years of operation), size of household (Household size), and the proportion of female workers hired in the total labor force (Women labor). First, household enterprises that have operated for a long period are often mature enterprises, which have a good knowledge about their businesses and have strong connections with suppliers, customers, and other players in the industry. Thus, household enterprises with a long history of operation are expected to be more productive compared with newly established enterprises. Second, it is usually easier for household enterprises to have more labor supply and get support if the household has more family members. We expect that the size of household has a positive effect on firm productivity. Third, evidence shows that women in Ghana are determined and hard-working (Dzisi, 2008). While Ghanaian women have lower levels of resources, technology, and education in general compared with their male counterparts, they learn to develop and utilize social relations and capital and combine them with their ability and flexibility in managing their household and enterprise (Kuada, 2009). It is also possible that women are more willing and able to work compared with men, since the latter have other businesses or obligations to deal with. Thus, hiring more female workers in the labor force is expected to improve the production efficiency of the household enterprise.

In addition, we control for the industry and the location where household enterprises operate. Specifically, we include three industry dummies that capture whether household enterprises have a business in trade (Trade), services other than trade (Other services), or sale of meals (Meal). It is expected that productivity is influenced by the industry of household enterprises. Further, we include five location dummies that capture whether household enterprises are located in Accra (Accra), other urban areas (Other urban), the rural coastal area (Rural coastal), the rural forest area (Rural forest), or the rural savannah area (Rural savannah). Household enterprises are expected to be more productive in urban areas, where there is more competition and larger market exposure.

Table 1A provides descriptive statistics for the variables we use in our empirical model. Table 1B presents the data sources and definitions of these variables in detail. Table 2 reports the correlations between the variables. More details of our data can be found in the Appendix. Specifically, Table A1 reports descriptive statistics of the variables used in the regression analysis for household enterprises that applied for formal credit. Table A2 reports descriptive statistics of the variables used in the regression analysis by gender. For each variable, a *t*-test is performed to test whether the mean of the variable differs significantly across male and female group. Results of the *t*-test are also reported. Tables A3, A4 report descriptive statistics of the variables used in the regression analysis by gender for household enterprises that applied for credit and for household enterprises that applied for credit and succeeded, respectively.

**Table 1A:** Descriptive statistics.

Variable	Obs	Mean	Std	Min	Max
Dependent variable					
Labor productivity	6.088	4.372	1.655	−0.916	11.695
Explanatory variable					
Not applied	6.959	0.895	0.307	0.000	1.000
Successful	6.959	0.085	0.279	0.000	1.000
Unsuccessful	6.959	0.020	0.141	0.000	1.000
Control variables					
*Household head characteristics*					
Age	6.962	53.250	13.970	17.000	97.000
Male	6.962	0.627	0.484	0.000	1.000
Female	6.962	0.373	0.484	0.000	1.000
Schooled	6.962	0.810	0.392	0.000	1.000
Not schooled	6.962	0.190	0.392	0.000	1.000
Married	6.962	0.648	0.478	0.000	1.000
Unmarried	6.962	0.352	0.478	0.000	1.000
*Enterprise characteristics*					
Years of operation	6.408	1.739	0.930	0.000	4.595
Women labor	6.959	0.711	0.435	0.000	1.000
Household size	6.962	4.072	3.225	1.000	28.00
*Enterprise location*					
Accra	6.962	0.011	0.103	0.000	1.000
Other urban	6.962	0.493	0.500	0.000	1.000
Rural coastal	6.962	0.069	0.254	0.000	1.000
Rural forest	6.962	0.152	0.359	0.000	1.000
Rural savannah	6.962	0.275	0.446	0.000	1.000
*Industry characteristics*					
Manufacturing	6.959	0.188	0.391	0.000	1.000
Trade	6.959	0.448	0.497	0.000	1.000
Other services	6.959	0.220	0.415	0.000	1.000
Meals	6.959	0.143	0.350	0.000	1.000

**Table 1B:** Data description and sources.

Variable	Definition	Source
**Dependent variable**		
Labor productivity (log)	Total value of sales divided by the number of workers employed by the enterprise.	S10, P(d); P(b), Q2
Explanatory variable		
Not applied	Not applied = 1, if a respondent answers “No” to “During the past 12 months, has this enterprise tried to get credit from banks, and other financial institutions?”; otherwise 0.	S10, P(a), Q15
Success	Success = 1, if a respondent answers “Yes, successfully” to “During the past 12 months, has this enterprise tried to get credit from banks, and other financial institutions?”; otherwise 0.	S10, P(a), Q15
Fail	Fail = 1, if a respondent answers “Yes, unsuccessfully” to “During the past 12 months, has this enterprise tried to get credit from banks, and other financial institutions?”; otherwise 0.	S10, P(a), Q15
Control variables		
*Entrepreneur characteristics*		
Age	Answer to “How old is (NAME)?.”	S1,Q5
Male	Male = 1, if a respondent answers “Male” to “SEX?”; otherwise 0.	S1,Q2
Female	Female = 1, if a respondent answers “Female” to “SEX?”; otherwise 0.	S1,Q2
Schooled	Schooled = 1, if a respondent answers “Yes” to “Has (NAME) ever attended school?”; otherwise 0.	S1, *p*(2), Q1
Married	Married = 1, if a respondent answers “Married” to “What is (NAME’S) present marital status?”; otherwise 0.	S1,Q6
*Enterprise characteristics*		
Years of operation	Answer to “How long has this enterprise been actively operating?.”	S10, P(a), Q7
Female participation	Share of female workers in total number of labor force.	S10, P(d); P(b), Q2
Household size	Sum of respondent, who answers “YES” to “Household member?.”	S1,Q24
*Enterprise location*		
Accra	Accra = 1, if the household enterprise is located in Accra area; otherwise 0.	GLSS 7
Other urban	Other urban = 1, if the household enterprise is located in other urban area; otherwise 0.	GLSS 7
Rural coastal	Rural coastal = 1, if the household enterprise is located in rural coastal area; otherwise 0.	GLSS 7
Rural forest	Rural forest = 1, if the household enterprise is located in rural forest area; otherwise 0.	GLSS 7
Rural savannah	Rural savannah = 1, if the household enterprise is located in rural savannah area; otherwise 0.	GLSS 7
*Industry characteristics*		GLSS 7
Trade	Trade = 1, if the enterprise is in the sector “Trade”; otherwise 0.	S10, P(d)
Other services	Other services = 1, if the enterprise is in the sector “services other than Trade (e.g., Running Transport, Hairdressing, Barbering, Repairing, Exchanging of foreign currency, Estate agents, etc.)”; otherwise 0.	S10, P(d)
Meals	Meals = 1, if the enterprise is in the sector “preparation and sales of meals”; otherwise 0.	S10, P(d)

**Table 2:** Pair-wise correlation matrix.

	[1]	[2]	[3]	[4]	[5]	[6]	[7]	[8]	[9]	[10]	[11]	[12]	[13]	[14]	[15]	[16]	[17]
[1] Labor productivity	1.00																
[2] Success	0.03	1.00															
[3] Fail	−0.02	−0.04	1.00														
[4] Male	−0.11	−0.01	0.00	1.00													
[5] Age	−0.08	−0.01	0.01	0.17	1.00												
[6] Married	−0.25	−0.01	0.01	0.54	0.44	1.00											
[7] Schooled	−0.01	−0.00	0.00	−0.01	0.02	−0.00	1.00										
[8] Years of operation	−0.00	0.02	0.01	−0.01	0.02	0.01	0.00	1.00									
[9] Women labor	0.07	−0.01	−0.02	−0.00	0.00	0.00	0.01	0.00	1.00								
[10] Household size	0.05	−0.01	−0.03	0.22	−0.09	−0.04	0.00	0.00	−0.00	1.00							
[11] Other urban	−0.17	0.00	0.01	−0.17	0.07	0.15	0.02	0.01	−0.03	−0.22	1.00						
[12] Rural coastal	0.36	0.01	0.02	−0.25	−0.15	−0.27	0.00	−0.01	0.01	−0.14	−0.27	1.00					
[13] Rural forest	−0.03	0.00	0.01	0.23	0.05	0.17	−0.03	0.01	0.01	−0.09	−0.42	−0.12	1.00				
[14] Rural savannah	−0.01	−0.02	−0.03	0.14	−0.02	−0.16	0.00	−0.01	0.02	0.40	−0.61	−0.17	−0.26	1.00			
[15] Trade	−0.01	0.06	0.00	−0.00	−0.00	0.02	−0.01	−0.08	0.00	−0.01	0.01	−0.00	0.00	−0.01	1.00		
[16] Other services	0.01	−0.09	−0.01	0.00	−0.00	−0.01	0.00	0.03	0.01	0.01	−0.01	0.00	−0.01	0.02	−0.48	1.00	
[17] Meals	−0.00	0.06	0.00	0.01	0.01	0.01	0.01	−0.05	−0.00	−0.02	−0.00	−0.01	0.02	−0.01	−0.37	−0.22	1.00

## Results

### Baseline analysis

The results of estimating the baseline model in Equation 1 is presented in Table 3. To start with, we regress labor productivity on enterprises’ use of formal credit only. The result in column (1) shows that household enterprises that applied and obtained credit, have a higher level of labor productivity, compared with those that did not apply. This is captured by the positive coefficient of Successful (0.153), which is significant at 10% level. It implies an expected productivity differential of 15.3% between enterprises that applied and got credit and those that did not apply, *ceteris paribus*. In column (2)–(4), we control for household head characteristics, enterprise characteristics, industry dummies, and location dummies, and redo the previous regression. The positive effect of obtaining formal credit on productivity is consistently found in the regressions. The coefficients of Successful remain positive and significant (0.146, 0.168, and 0.151 respectively) at 10% level. Overall, we expect a productivity differential of around 15% between enterprises that applied and got credit and those that did not apply, *ceteris paribus*.

**Table 3:** Formal finance and firm productivity: full sample analysis.

Dependent variable: labor productivity	(1)	(2)	(3)	(4)
Successful	0.153^*^	0.146^*^	0.168^**^	0.151^*^
	(0.080)	(0.078)	(0.082)	(0.080)
Unsuccessful	−0.199	−0.168	−0.133	−0.185
	(0.154)	(0.148)	(0.151)	(0.148)
Male		0.078	0.014	0.156^***^
		(0.048)	(0.051)	(0.054)
Age		0.006^***^	0.006^***^	0.007^***^
		(0.002)	(0.002)	(0.002)
Married		−0.980^***^	−0.938^***^	−0.685^***^
		(0.055)	(0.058)	(0.061)
Schooled		0.043	0.046	0.038
		(0.053)	(0.055)	(0.052)
Years of operation			0.001	0.002
			(0.023)	(0.022)
Women labor			0.264^***^	0.255^***^
			(0.049)	(0.047)
Household size			0.025^***^	0.050^***^
			(0.006)	(0.006)
Trade				−0.019
				(0.055)
Other services				0.021
				(0.062)
Meals				−0.018
				(0.073)
Other urban				−0.289
				(0.217)
Rural coastal				1.820^***^
				(0.238)
Rural forest				−0.208
				(0.223)
Rural savannah				−0.345
				(0.219)
Constant	4.363^***^	4.654^***^	4.369^***^	4.084^***^
	(0.022)	(0.099)	(0.126)	(0.240)
Observations	6,086	6,086	5,608	5,608
*R* ^2^	0.001	0.063	0.069	0.166

This table presents the results of estimating the effect of formal finance on firm productivity based on the full sample. All specifications are estimated using OLS. Robust standard errors in parentheses, ^***^significant at 1% level, ^**^significant at 5% level, ^*^significant at 10% level.

When it comes to the control variables, we find that household head’s age (Age) and marital status (Married) explain the level of enterprise labor productivity. First, labor productivity is found higher in household enterprises, whose head is older. This suggests that senior household heads have more business experiences and management skills and are more likely to achieve business success. Second, labor productivity is found higher in household enterprises, whose head is unmarried. This supports the view that married household heads may have to bear more family obligations, which leave them less time and effort that can be put in their enterprises and, thus, hinder them from achieving as good business performances as unmarried household heads can otherwise do.

Furthermore, enterprises that have a larger size of household (Household size) and hire more female employees (Women labor) are expected to be more labor productive. Specifically, we interpret household size as an indicator that captures the overall labor and resource endowment of the enterprise. Hence, household enterprises having more resources are more likely to achieve business success. The extent of female participation may reflect the level of productive labor input of production for household enterprises. As is mentioned in Section “Literature review”, there is evidence showing that indigenous women in Ghana are confident, determined, and hard-working (e.g., Dzisi, 2008), which may help to explain our finding that household enterprises that hire more female employees are more likely to be labor productive. Finally, our result suggests that household enterprises located in the rural costal area tend to be more productive compared with those located in Accra, i.e., our reference group. Overall, the empirical analyses we have performed so far support our first hypothesis that obtaining formal credit has a positive effect on labor productivity of non-farm household enterprises in Ghana.

As is discussed in Section “Literature review”, the effect of formal credit on firm performance may differ by the gender of entrepreneurs, as female entrepreneurs are bounded with more severe social and economic constraints. In Ghana, according to the GLSS 7 women dominate (58.4%) the non-farm household enterprises and this cuts across the major sectors of operation, manufacturing (65.7%), trading (72%) and 57.6% in other industries. Female dominance is a stylized feature of non-farm household enterprise in Ghana as can be seen from previous GLSS results and is in half of the cases either the wife (in a male-headed household) or in the other half, herself as head in a female-headed household who is responsible for the enterprise. As is noted by Ackah (2013), in Ghana women headed households tend to move away from wage employment toward self-employment and thus more engaged in non-farm household enterprises.

To test gender difference in the effect of formal credit on firm performance, we further conduct a subsample analysis, in which we redo the regressions we have performed in Table 4 for male-headed and female-headed household enterprises separately. Table 4 reports the estimation results. The message is that formal credit seems to enhance labor productivity only for household enterprises domiciled in male-headed households, but not for those domiciled in female-headed households ones. In the sub-sample of non-farm household enterprises in male-headed households [column (1)–(4)], the coefficients of Successful are always positive and significant (0.195, 0.188, 0.242, and 0.245 respectively). Overall, we expect a productivity differential of around 25%, *ceteris paribus*, between enterprises that got credit and those that did not apply for credit in the male-headed sub-sample. However, in the sub-sample of female-headed enterprises the coefficients of Successful are never significant [column (5)–(8)]. This is in line with the findings presented by De Mel et al. (2008) and Fafchamps et al. (2014). Of the control variables, household size (Household size), household head’s age (Age) and marital status (Married), the share of female employees in the labor force (Women labor) are again found to have a strong explanatory power on household enterprise productivity. In general, our results support our second hypothesis that the effect of formal credit on firm performance is weaker for female-headed enterprises.

**Table 4:** Formal finance and firm productivity: full sample analysis by gender.

Dependent variable: labor productivity	(1)	(2)	(3)	(4)	(5)	(6)	(7)	(8)
	Male-headed household enterprises				Female-headed household enterprises			
Successful	0.195^**^	0.188^**^	0.242^***^	0.245^***^	0.075	0.016	−0.009	−0.074
	(0.083)	(0.081)	(0.086)	(0.086)	(0.164)	(0.155)	(0.159)	(0.148)
Unsuccessful	−0.282	−0.267	−0.260	−0.269	−0.057	0.003	0.139	0.010
	(0.179)	(0.179)	(0.180)	(0.180)	(0.277)	(0.268)	(0.279)	(0.286)
Age		−0.011^***^	−0.009^***^	−0.009^***^		0.038^***^	0.035^***^	0.034^***^
		(0.002)	(0.002)	(0.002)		(0.004)	(0.004)	(0.004)
Married		−0.385^***^	−0.280^***^	−0.292^***^		−1.818^***^	−1.910^***^	−1.413^***^
		(0.064)	(0.082)	(0.083)		(0.101)	(0.118)	(0.130)
Schooled		0.007	0.001	−0.000		0.058	0.077	0.053
		(0.054)	(0.056)	(0.056)		(0.109)	(0.114)	(0.101)
Years of operation			−0.021	−0.021			0.003	0.017
			(0.023)	(0.023)			(0.046)	(0.042)
Women labor			0.243^***^	0.243^***^			0.314^***^	0.291^***^
			(0.049)	(0.049)			(0.100)	(0.092)
Household size			0.028^***^	0.033^***^			0.078^***^	0.102^***^
			(0.008)	(0.008)			(0.015)	(0.015)
Trade				0.007				−0.053
				(0.059)				(0.105)
Other services				0.118^*^				−0.126
				(0.065)				(0.118)
Meals				0.058				−0.232
				(0.075)				(0.146)
Other urban				−0.108				0.187
				(0.244)				(0.371)
Rural coastal				−0.039				2.577^***^
				(0.310)				(0.380)
Rural forest				−0.048				−0.232
				(0.250)				(0.412)
Rural savannah				−0.186				−0.128
				(0.245)				(0.392)
Constant	4.229^***^	5.157^***^	4.711^***^	4.771^***^	4.597^***^	3.369^***^	3.081^***^	2.416^***^
	(0.021)	(0.098)	(0.158)	(0.272)	(0.048)	(0.182)	(0.221)	(0.434)
Observations	3,874	3,874	3,575	3,575	2,212	2,212	2,033	2,033
*R* ^2^	0.003	0.040	0.051	0.054	0.000	0.125	0.129	0.304

This table presents the results of estimating the effect of formal finance on firm productivity based on the full sample. Column (1)–(4) shows the results for male-headed household enterprises. Column (5)–(8) shows the results for female-headed household enterprises. All specifications are estimated using OLS. Robust standard errors in parentheses, ^***^significant at 1% level, ^**^significant at 5% level, ^*^significant at 10% level.

### Robustness checks

To test the robustness our results, we remove household enterprises that have not applied for credit from the previous sample. The idea is that household enterprises may choose to exclude themselves from formal finance, because they believe that they do not meet the requirements so that they will not succeed in obtaining credit anyway, even if they apply for credit, or because they simply do not have the need for financial services. To address the role of selection bias in explaining firm productivity, we redo the baseline regressions on this smaller sample, which is composed of household enterprises that have applied for formal credit only. In general, the robustness checks yield consistent results with what we have found before. First, the results from Table 5 shows that household enterprises, which have tried to apply for formal credit and finally succeeded, have a higher level of labor productivity, compared with those that have applied but failed. This is captured by the positive and significant coefficients of Successful. Second, when we conduct a subsample analysis in Table 6, we find again that the positive effect of formal credit on productivity appears in male-headed enterprises only, but not in female-headed ones.

**Table 5:** Formal finance and firm productivity: small sample analysis.

Dependent variable: labor productivity	(1)	(2)	(3)	(4)
Success	0.352^**^	0.304^*^	0.289^*^	0.344^**^
	(0.171)	(0.167)	(0.172)	(0.170)
Male		0.128	0.156	0.247
		(0.158)	(0.165)	(0.170)
Age		−0.004	−0.007	−0.004
		(0.006)	(0.006)	(0.006)
Married		−0.856^***^	−0.859^***^	−0.694^***^
		(0.180)	(0.186)	(0.198)
Schooled		0.226	0.191	0.216
		(0.172)	(0.176)	(0.174)
Years of operation			−0.047	−0.031
			(0.075)	(0.071)
Women labor			0.530^***^	0.486^***^
			(0.162)	(0.158)
Household size			0.008	0.027
			(0.021)	(0.022)
Trade				−0.024
				(0.214)
Other services				0.312
				(0.252)
Meals				−0.033
				(0.241)
Other urban				−1.723^**^
				(0.745)
Rural coastal				−0.097
				(0.791)
Rural forest				−1.493^*^
				(0.765)
Rural savannah				−1.712^**^
				(0.755)
Constant	4.164^***^	4.881^***^	4.721^***^	5.770^***^
	(0.153)	(0.364)	(0.457)	(0.841)
Observations	635	635	577	577
*R* ^2^	0.006	0.065	0.087	0.160

This table presents the results of estimating the effect of formal finance on firm productivity based on the small sample, which consists of household enterprises that applied for formal credit only. All specifications are estimated using OLS. Robust standard errors in parentheses, ^***^significant at 1% level, ^**^significant at 5% level, ^*^significant at 10% level.

**Table 6:** Formal finance and firm productivity: small sample analysis by gender.

Dependent variable: labor productivity	(1)	(2)	(3)	(4)	(5)	(6)	(7)	(8)
	Male-headed household enterprises				Female-headed household enterprises			
Success	0.477^**^	0.450^**^	0.510^**^	0.509^**^	0.132	−0.008	−0.248	−0.229
	(0.195)	(0.199)	(0.202)	(0.205)	(0.316)	(0.301)	(0.328)	(0.344)
Age		−0.018^***^	−0.018^***^	−0.014^**^		0.024^*^	0.015	0.020
		(0.006)	(0.006)	(0.006)		(0.013)	(0.014)	(0.015)
Married		−0.210	−0.187	−0.290		−1.719^***^	−1.855^***^	−1.651^***^
		(0.230)	(0.271)	(0.275)		(0.315)	(0.358)	(0.400)
Schooled		0.165	0.160	0.155		0.312	0.371	0.434
		(0.194)	(0.205)	(0.201)		(0.318)	(0.335)	(0.341)
Years of operation			−0.043	−0.004			−0.064	−0.079
			(0.080)	(0.080)			(0.141)	(0.129)
Women labor			0.244	0.220			1.136^***^	1.115^***^
			(0.180)	(0.176)			(0.335)	(0.332)
Household size			0.016	0.020			0.049	0.095^**^
			(0.028)	(0.028)			(0.047)	(0.047)
Trade				0.144				−0.140
				(0.263)				(0.367)
Other services				0.502^*^				0.043
				(0.293)				(0.458)
Meals				0.226				−0.217
				(0.278)				(0.444)
Other urban				−1.934^**^				1.979^***^
				(0.756)				(0.549)
Rural coastal				−1.118				3.572^***^
				(0.860)				(0.628)
Rural forest				−1.628^**^				0.607
				(0.776)				(0.648)
Rural savannah				−1.875^**^				1.592^***^
				(0.768)				(0.572)
Constant	3.947^***^	5.092^***^	4.878^***^	6.327^***^	4.540^***^	3.990^***^	3.863^***^	1.313
	(0.178)	(0.441)	(0.608)	(0.922)	(0.274)	(0.643)	(0.777)	(1.161)
Observations	400	400	363	363	235	235	214	214
*R* ^2^	0.016	0.060	0.076	0.117	0.001	0.118	0.171	0.283

This table presents the results of estimating the effect of formal finance on firm productivity based on the small sample, which consists of household enterprises that applied for formal credit only. Column (1)–(4) shows the results for male-headed household enterprises. Column (5)–(8) shows the results for female-headed household enterprises. All specifications are estimated using OLS. Robust standard errors in parentheses, ^***^significant at 1% level, ^**^significant at 5% level, ^*^significant at 10% level.

**Figure 3:**
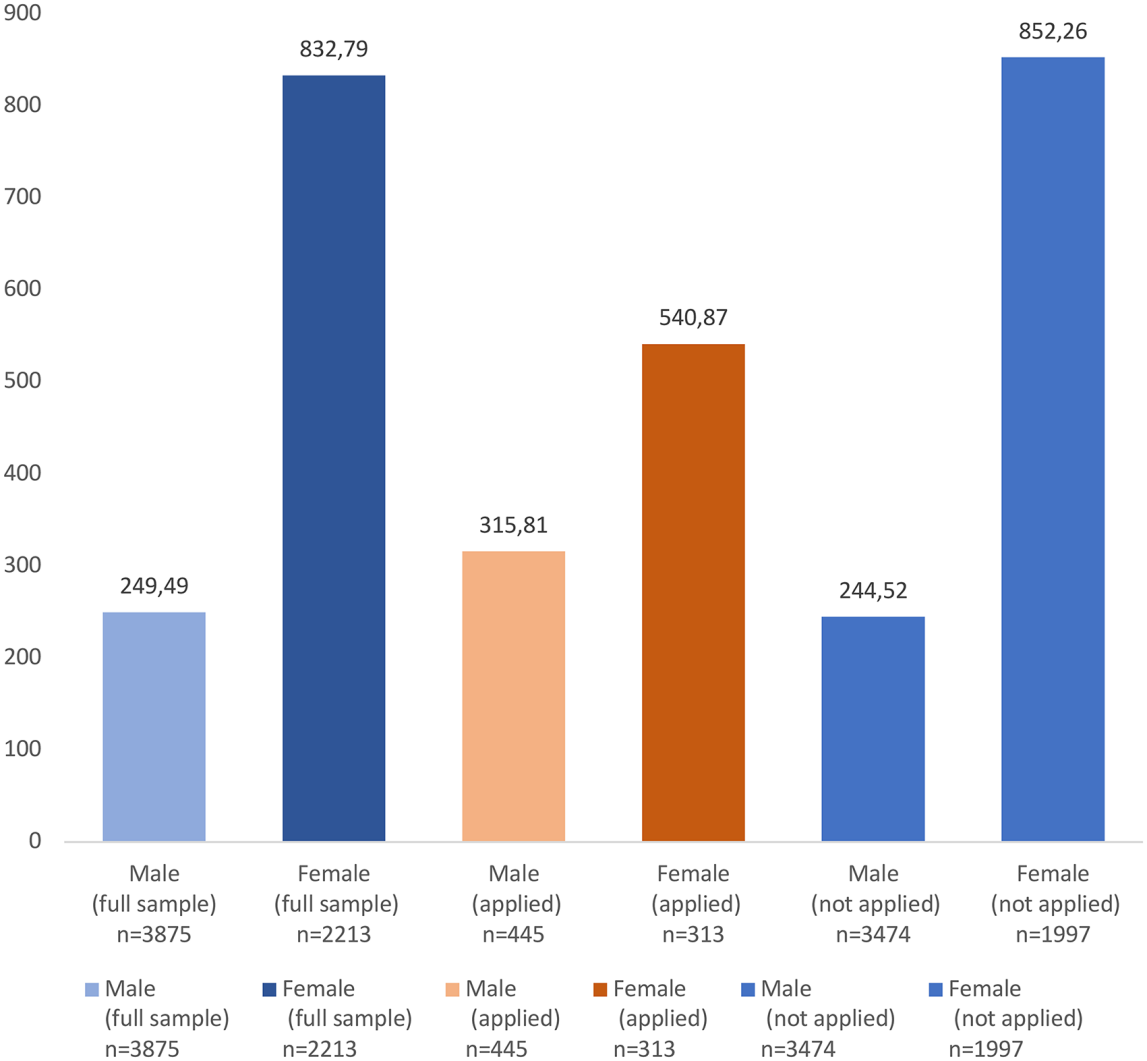
Labor productivity by the gender of household head (GH¢ per employee).

We provide two possible explanations. First, diminishing marginal returns to capital may be at work. As is illustrated in Figure 3, female-headed enterprises are far more productive than male-headed enterprises (832.79 GH¢ per employee versus 249.49 GH¢ per employee) in our sample. This gender difference in productivity holds in both the subsample of enterprises that have applied for formal credit (540.87 GH¢ per employee versus 315.81 GH¢ per employee) and the subsample of enterprises that have not applied for formal credit (852.26 GH¢ per employee versus 244.52 GH¢ per employee). Given that female-headed household enterprises, on average, exhibit a much higher level of labor productivity than male-headed enterprises, using formal credit among female-headed household enterprises may have little effect on further improving labor productivity. Second, and related to the previous remark, female-headed enterprises may still need to improve complementary inputs, such as business knowledge, management skills, and financial literacy, to reap the full benefits of using formal credit. In fact, the importance of having complementary inputs have been highlighted in the microfinance literature. The main idea is that a lack of access to credit may not be the key impediment to firm growth. Other factors, such as business know-how and financial knowledge, are complementary to microcredit. That is why many microfinance institutions have included business and financial literacy trainings into their lending strategy, often known as the “microfinance-plus” strategy.^12^

We have tried to test for the potential effect of complementary inputs using available data from the survey, including household heads’ marital status (Married), education (Schooled), business experience (Years of operation), household size (Household size), and share of female workers employed in the labor force (Women labor), all of which are assumed to indicate some of the key constraints faced by female entrepreneurs in the literature (e.g., Bardasi et al., 2011). We are interested to examine whether the effect of formal credit on firm productivity depends on whether a household head is married, has ever gone to school, has at least one-year experience of running the enterprise, has a large household (has more family members), and hires female workers. Unfortunately, we do not find any significant results with respect to either the direct effect of formal credit, or the conditioning effect of the above complementary factors in both male-and female-headed household enterprises, except for the share of female workers hired. Estimation results are reported in Tables 7, 8.

**Table 7:** Formal finance and firm productivity: full sample analysis by gender, including interaction terms.

Dependent variable: labor productivity	(1)	(2)	(3)	(4)	(5)	(6)	(7)	(8)	(9)	(10)	(11)	(12)
	Male-headed household enterprises						Female-headed household enterprises					
Successful	0.220	−0.142	0.245	0.285^*^	0.305^**^	0.083	−0.045	−0.637	0.237	−1.002^***^	−0.093	0.041
	(0.223)	(0.278)	(0.188)	(0.163)	(0.150)	(0.287)	(0.196)	(0.450)	(0.292)	(0.295)	(0.228)	(0.360)
Unsuccessful	−0.081	0.072	−0.179	−0.442	−0.371	−0.535	0.052	0.597	0.390	1.080^**^	0.127	−0.424
	(0.725)	(0.591)	(0.371)	(0.425)	(0.317)	(0.393)	(0.432)	(1.168)	(0.665)	(0.461)	(0.426)	(0.748)
Age	−0.009^***^	−0.009^***^	−0.009^***^	−0.009^***^	−0.009^***^	−0.009^***^	0.034^***^	0.034^***^	0.034^***^	0.033^***^	0.034^***^	0.034^***^
	(0.002)	(0.002)	(0.002)	(0.002)	(0.002)	(0.002)	(0.004)	(0.004)	(0.004)	(0.004)	(0.004)	(0.004)
Married	−0.291^***^	−0.293^***^	−0.292^***^	−0.292^***^	−0.293^***^	−0.295^***^	−1.403^***^	−1.414^***^	−1.411^***^	−1.398^***^	−1.414^***^	−1.415^***^
	(0.085)	(0.083)	(0.083)	(0.083)	(0.083)	(0.083)	(0.134)	(0.130)	(0.130)	(0.130)	(0.130)	(0.131)
Schooled	−0.001	−0.021	−0.000	0.000	−0.002	−0.000	0.053	0.021	0.049	0.072	0.053	0.056
	(0.056)	(0.057)	(0.056)	(0.056)	(0.056)	(0.056)	(0.101)	(0.106)	(0.101)	(0.100)	(0.101)	(0.101)
Years of operation	−0.021	−0.021	−0.020	−0.022	−0.021	−0.020	0.017	0.016	0.037	0.026	0.018	0.022
	(0.023)	(0.023)	(0.023)	(0.023)	(0.023)	(0.023)	(0.042)	(0.042)	(0.045)	(0.042)	(0.042)	(0.042)
Women labor	0.244^***^	0.241^***^	0.244^***^	0.243^***^	0.243^***^	0.243^***^	0.293^***^	0.296^***^	0.287^***^	0.221^**^	0.293^***^	0.293^***^
	(0.049)	(0.049)	(0.049)	(0.051)	(0.049)	(0.049)	(0.093)	(0.092)	(0.092)	(0.097)	(0.092)	(0.092)
Household size	0.033^***^	0.033^***^	0.033^***^	0.033^***^	0.033^***^	0.033^***^	0.102^***^	0.101^***^	0.102^***^	0.103^***^	0.102^***^	0.102^***^
	(0.008)	(0.008)	(0.008)	(0.008)	(0.009)	(0.008)	(0.015)	(0.015)	(0.015)	(0.015)	(0.016)	(0.015)
Trade	0.006	0.006	0.007	0.006	0.007	−0.006	−0.053	−0.051	−0.052	−0.067	−0.053	−0.051
	(0.058)	(0.059)	(0.059)	(0.059)	(0.059)	(0.059)	(0.105)	(0.105)	(0.105)	(0.104)	(0.105)	(0.110)
Other services	0.118^*^	0.118^*^	0.118^*^	0.117^*^	0.118^*^	0.089	−0.126	−0.123	−0.133	−0.133	−0.127	−0.142
	(0.065)	(0.065)	(0.065)	(0.065)	(0.065)	(0.067)	(0.118)	(0.118)	(0.118)	(0.117)	(0.118)	(0.122)
Meals	0.059	0.057	0.058	0.056	0.060	0.046	−0.231	−0.232	−0.230	−0.217	−0.232	−0.221
	(0.075)	(0.075)	(0.075)	(0.075)	(0.075)	(0.079)	(0.146)	(0.146)	(0.146)	(0.145)	(0.146)	(0.156)
Other urban	−0.109	−0.109	−0.108	−0.108	−0.107	−0.107	0.187	0.193	0.189	0.144	0.179	0.189
	(0.244)	(0.244)	(0.243)	(0.244)	(0.244)	(0.243)	(0.372)	(0.371)	(0.371)	(0.367)	(0.370)	(0.372)
Rural coastal	−0.038	−0.039	−0.040	−0.038	−0.040	−0.046	2.577^***^	2.585^***^	2.581^***^	2.567^***^	2.568^***^	2.585^***^
	(0.310)	(0.310)	(0.310)	(0.311)	(0.311)	(0.309)	(0.381)	(0.380)	(0.380)	(0.375)	(0.379)	(0.381)
Rural forest	−0.048	−0.049	−0.048	−0.047	−0.046	−0.044	−0.231	−0.224	−0.234	−0.288	−0.241	−0.231
	(0.250)	(0.249)	(0.249)	(0.250)	(0.250)	(0.249)	(0.413)	(0.412)	(0.412)	(0.408)	(0.411)	(0.413)
Rural savannah	−0.187	−0.188	−0.186	−0.186	−0.184	−0.183	−0.129	−0.125	−0.126	−0.164	−0.137	−0.132
	(0.245)	(0.245)	(0.244)	(0.245)	(0.245)	(0.244)	(0.393)	(0.392)	(0.392)	(0.387)	(0.391)	(0.393)
Successful × Married	0.030						−0.083					
	(0.241)						(0.286)					
Unsuccessful × Married	−0.217						−0.096					
	(0.746)						(0.556)					
Successful × Schooled		0.325						0.475				
		(0.226)						(0.342)				
Unsuccessful × Schooled		−0.279						−0.524				
		(0.489)						(1.052)				
Successful × Years of operation			−0.000						−0.178			
			(0.089)						(0.146)			
Unsuccessful × Years of operation			−0.051						−0.192			
			(0.208)						(0.293)			
Successful × Women labor				−0.058						1.295^***^		
				(0.195)						(0.338)		
Unsuccessful × Women Labor				0.261						−1.667^***^		
				(0.465)						(0.584)		
Successful × Household size					−0.013						0.007	
					(0.023)						(0.044)	
Unsuccessful × Household size					0.028						−0.041	
					(0.059)						(0.089)	
Successful × Trade						0.164						−0.133
						(0.310)						(0.417)
Unsuccessful× Trade						0.363						0.222
						(0.366)						(0.497)
Successful × Other services						0.132						−0.310
						(0.336)						(0.495)
Unsuccessful × Other services						0.176						0.384
						(0.511)						(0.849)
Successful × Meal						0.650						0.516
						(0.507)						(0.891)
Unsuccessful × Meal						0.381						1.516^*^
						(0.427)						(0.899)
Constant	4.766^***^	4.779^***^	4.770^***^	4.776^***^	4.767^***^	4.787^***^	2.411^***^	2.403^***^	2.390^***^	2.520^***^	2.423^***^	2.407^***^
	(0.272)	(0.272)	(0.271)	(0.272)	(0.273)	(0.271)	(0.435)	(0.434)	(0.434)	(0.431)	(0.433)	(0.436)
Observations	3,575	3,575	3,575	3,575	3,575	3,575	2,033	2,033	2,033	2,033	2,033	2,033
*R* ^2^	0.054	0.055	0.054	0.054	0.054	0.055	0.304	0.305	0.305	0.312	0.304	0.305

This table presents the results of estimating the effect of formal finance on firm productivity based on the full sample. Column (1)–(6) shows the results for male-headed household enterprises. Column (7)–(12) shows the results for female-headed household enterprises. All specifications are estimated using OLS. Robust standard errors in parentheses, ^***^significant at 1% level, ^**^significant at 5% level, ^*^significant at 10% level.

**Table 8:** Formal finance and firm productivity: small sample analysis by gender, including interaction terms.

Dependent variable: labor productivity	(1)	(2)	(3)	(4)	(5)	(6)	(7)	(8)	(9)	(10)	(11)	(12)
	Male-headed household enterprises						Female-headed household enterprises					
Success	0.398	−0.290	0.355	0.801^*^	0.606^*^	0.598	−0.313	−1.580	−0.286	−2.089^***^	−0.416	0.269
	(0.750)	(0.642)	(0.420)	(0.471)	(0.358)	(0.497)	(0.510)	(1.229)	(0.773)	(0.550)	(0.518)	(0.891)
Age	−0.014^**^	−0.014^**^	−0.014^**^	−0.015^**^	−0.014^**^	−0.015^**^	0.020	0.022	0.020	0.017	0.020	0.020
	(0.006)	(0.006)	(0.006)	(0.006)	(0.006)	(0.006)	(0.015)	(0.014)	(0.015)	(0.014)	(0.015)	(0.015)
Married	−0.396	−0.288	−0.287	−0.288	−0.297	−0.297	−1.805^***^	−1.690^***^	−1.648^***^	−1.451^***^	−1.664^***^	−1.682^***^
	(0.767)	(0.274)	(0.275)	(0.275)	(0.275)	(0.271)	(0.653)	(0.393)	(0.402)	(0.400)	(0.406)	(0.407)
Schooled	0.153	−0.362	0.155	0.167	0.146	0.165	0.437	−0.577	0.430	0.555^*^	0.429	0.451
	(0.203)	(0.469)	(0.201)	(0.202)	(0.201)	(0.202)	(0.343)	(1.033)	(0.342)	(0.318)	(0.342)	(0.341)
Years of operation	−0.003	−0.004	−0.075	−0.011	−0.003	−0.009	−0.075	−0.097	−0.103	−0.051	−0.074	−0.055
	(0.081)	(0.080)	(0.215)	(0.082)	(0.080)	(0.081)	(0.130)	(0.130)	(0.321)	(0.125)	(0.129)	(0.129)
Women labor	0.220	0.202	0.224	0.563	0.220	0.207	1.124^***^	1.127^***^	1.116^***^	−1.197^*^	1.133^***^	1.149^***^
	(0.176)	(0.177)	(0.178)	(0.470)	(0.176)	(0.177)	(0.342)	(0.331)	(0.334)	(0.628)	(0.332)	(0.338)
Household size	0.021	0.024	0.020	0.019	0.042	0.020	0.093^*^	0.094^**^	0.095^**^	0.108^**^	0.039	0.102^**^
	(0.028)	(0.028)	(0.028)	(0.028)	(0.062)	(0.028)	(0.048)	(0.047)	(0.047)	(0.048)	(0.097)	(0.048)
Trade	0.137	0.133	0.143	0.120	0.149	0.092	−0.156	−0.086	−0.143	−0.365	−0.145	0.208
	(0.258)	(0.259)	(0.263)	(0.267)	(0.262)	(0.522)	(0.371)	(0.362)	(0.366)	(0.355)	(0.371)	(0.930)
Other services	0.499^*^	0.500^*^	0.504^*^	0.477	0.505^*^	0.433	0.024	0.097	0.038	−0.084	0.039	−0.023
	(0.291)	(0.288)	(0.293)	(0.291)	(0.294)	(0.355)	(0.467)	(0.453)	(0.463)	(0.447)	(0.461)	(0.504)
Meals	0.223	0.202	0.229	0.194	0.235	0.563	−0.223	−0.187	−0.222	−0.340	−0.222	1.349
	(0.277)	(0.275)	(0.278)	(0.282)	(0.278)	(0.446)	(0.448)	(0.444)	(0.443)	(0.429)	(0.446)	(1.093)
Other urban	−1.934^**^	−1.940^**^	−1.940^**^	−1.937^***^	−1.930^**^	−1.948^**^	2.044^***^	2.152^***^	1.972^***^	0.924	1.760^***^	2.014^***^
	(0.755)	(0.749)	(0.763)	(0.746)	(0.760)	(0.771)	(0.610)	(0.534)	(0.554)	(0.616)	(0.569)	(0.628)
Rural coastal	−1.111	−1.101	−1.125	−1.100	−1.124	−1.146	3.634^***^	3.745^***^	3.564^***^	2.828^***^	3.346^***^	3.641^***^
	(0.860)	(0.862)	(0.867)	(0.854)	(0.864)	(0.877)	(0.697)	(0.588)	(0.630)	(0.653)	(0.650)	(0.731)
Rural forest	−1.628^**^	−1.633^**^	−1.632^**^	−1.620^**^	−1.623^**^	−1.629^**^	0.678	0.744	0.600	−0.491	0.367	0.592
	(0.775)	(0.770)	(0.783)	(0.766)	(0.780)	(0.790)	(0.713)	(0.641)	(0.651)	(0.710)	(0.687)	(0.721)
Rural savannah	−1.877^**^	−1.883^**^	−1.881^**^	−1.882^**^	−1.867^**^	−1.882^**^	1.654^**^	1.711^***^	1.582^***^	0.648	1.378^**^	1.569^**^
	(0.767)	(0.762)	(0.776)	(0.758)	(0.773)	(0.783)	(0.655)	(0.566)	(0.578)	(0.555)	(0.560)	(0.693)
Successful × Married	0.129						0.198					
	(0.779)						(0.691)					
Successful × Schooled		0.658						1.181				
		(0.518)						(1.077)				
Successful × Years of operation			0.087						0.029			
			(0.230)						(0.350)			
Successful × Women labor				−0.432						2.910^***^		
				(0.510)						(0.696)		
Successful × Household size					−0.026						0.067	
					(0.064)						(0.106)	
Successful × Trade						0.043						−0.467
						(0.601)						(1.003)
Successful × Other services						−0.412						−1.807
						(0.550)						(1.171)
Successful × Meal						0.220						0.335
						(0.619)						(1.152)
Constant	6.406^***^	6.463^***^	6.455^***^	6.163^***^	6.243^***^	6.326^***^	1.332	1.191	1.376	3.856^***^	1.676	0.820
	(1.106)	(0.926)	(0.962)	(0.961)	(0.961)	(0.963)	(1.167)	(1.127)	(1.354)	(1.284)	(1.197)	(1.576)
Observations	363	363	363	363	363	363	214	214	214	214	214	214
*R* ^2^	0.117	0.122	0.118	0.120	0.118	0.119	0.283	0.288	0.283	0.337	0.284	0.291

This table presents the results of estimating the effect of formal finance on firm productivity based on the small sample, which consists of household enterprises that applied for formal credit only. Column (1)–(6) shows the results for male-headed household enterprises. Column (7)–(12) shows the results for female-headed household enterprises. All specifications are estimated using OLS. Robust standard errors in parentheses, ^***^significant at 1% level, ^**^significant at 5% level, ^*^significant at 10% level.

Plausible explanations on the insignificant results can be twofold. First, the variables we use to measure the complementary factors are not accurate. To illustrate, marital status, i.e., whether household head is married or not, only roughly captures to what extent the head is occupied for taking care of family or domestic obligations. Education, indicated by whether the household head has attended school, is not sufficient to indicate the level of knowledge the head has acquired. The number of years when the enterprise has been operating may not be a precise indicator of household head’s business experiences. Since GLSS 7 only provides us with these (proxy) indicators that we can explore, we are not able to test potential conditional effects further. Second, and also related to the previous remark, the conditions based on which credit may potentially enhance labor productivity of female-headed household enterprises are not covered in GLSS7. For example, there is evidence showing that female household heads tend to have the ability to better manage household resources and generate further welfare benefits for their households, e.g., food security, (Dzanku, 2019). Also, Ghanaian women learn to develop and use social relations and combine them with their capability of managing their households and enterprises to (Kuada, 2009). However, these complementary capabilities are difficult to be defined and measured in the first place, and a lack of these information in GLSS 7 means that we are not able to directly test the role of these capabilities in the effect of formal credit on firm productivity.

## Conclusion

In this study, we investigate the effect of obtaining credit from bank and other formal financial institutions on labor productivity of non-farm household enterprises in Ghana. Based on a sample of 6,959 non-farm household enterprises collected from Ghana Living Standard Survey 2016/2017, our empirical analysis has established two main findings. First, formal credit has a positive and significant effect on labor productivity of household enterprises. This is in line with the literature that highlights the indispensable role of finance in firm growth and firm performance (e.g., Ayyagari et al., 2008, 2010). Therefore, policies aimed at promoting the use of formal finance in Ghana are desired to support non-farm household enterprises to improve their firm performance. Second, we find that the positive effect of formal credit only appears in male-headed household enterprises, but not in female-headed ones. In fact, the gender gap in the link between finance and firm performance is also found in other country studies, where randomized control trials are employed to examine the impact of financial capital, together with other interventions, on the business outcomes of microenterprises, e.g., in Sri Lanka (De Mel et al., 2008, 2009), Ghana (Fafchamps et al., 2014), and Tanzania (Berge et al., 2015).

Nevertheless, two questions remain unclear: (1) why does the credit effect disappear for female-headed household enterprises; and (2) under what conditions may formal credit enhance labor productivity of female-headed household enterprises? We suspect that for credit to generate productivity-enhancing effect the head of household enterprises should possess certain skills, knowledge, experiences, and capabilities. Since women in general, less endowed with these conditional factors compared with men, the influence of formal credit on productivity may be too small to be significant. We have tried to test for the potential effect of complementary inputs using available data from GLSS7. Unfortunately, we do not find any significant results with respect to either the direct effect of formal credit, or the conditioning effect of the above complementary factors, except for the share of female workers employed.

One direction for future research may be to further explore the complementary factors that may potentially moderate the effect of formal finance on labor productivity of non-farm household enterprises in Ghana. However, this will have to rely on future waves of Ghanaian Living Standard Survey, which provide researchers with deeper information and more accurate measures to capture, e.g., household head’s formal education, informal education, such as business training, apprenticeship, financial literacy, and the degree to which he/she has to take care of responsibilities that are not directly associated with their enterprise.^13^ Besides, future research may look into potential social and psychological factors. The intuition is that the effect of formal credit on firm productivity may depend on household heads’ endowment, e.g., his/her entrepreneurial ambition, commitment to business, preference of risks, acceptability of new ideas and concepts, conscientiousness, and inter-personal skills, to name a few. In fact, these social and psychological factors may be able to explain the heterogeneity in the productivity-enhancing effect of credit between male and female-headed enterprises, which we are not able to answer in this study. Once again, this will depend on future waves of the Ghanaian Living Standard Surveys, which can incorporate relevant social and psychological questions into the questionnaire.

## Data availability statement

The raw data supporting the conclusions of this article will be made available by the authors, without undue reservation.

## Author contributions

CA contributed to conception and design of the study. YP organized the database, performed the statistical analysis, and wrote the first draft of the manuscript. All authors contributed to the article and approved the submitted version.

## Conflict of interest

The authors declare that the research was conducted in the absence of any commercial or financial relationships that could be construed as a potential conflict of interest.

## Publisher’s note

All claims expressed in this article are solely those of the authors and do not necessarily represent those of their affiliated organizations, or those of the publisher, the editors and the reviewers. Any product that may be evaluated in this article, or claim that may be made by its manufacturer, is not guaranteed or endorsed by the publisher.

## Footnotes

^1^Source: www.un.org/sustainabledevelopment/gender-equality

^2^Source: www.ilo.org/global/about-the-ilo/newsroom/news/WCMS_626831/lang--en/index.htm

^3^See Carreira and Silva (2010) for a review of the literature on firms’ financial constraints.

^4^Beck et al. (2005) measure financing constraints as follows. The variable financing barrier takes a value between 1 and 4, where 1 indicates no obstacle and 4 indicates major obstacle, based on the survey question “How problematic is financing for the operation and growth of your business: no obstacle (1), a minor obstacle (2), a moderate obstacle (3), or a major obstacle (4)?.” Data is drawn from the World Business Environment Survey conducted by the World Bank.

^5^Formally, access to finance is defined as "the availability of a supply of reasonable quality financial services at reasonable costs" (Claessens, 2006, p.210). It is noteworthy that access to finance is not the same as use of finance. Access refers to the supply of financial services, while use is a combined outcome of demand for and supply of finance. That is, firms that have access to finance may voluntarily choose not to use finance because they do not need finance, or because they know they will be rejected by financial institutions to use finance. In this study, we focus on use of finance. Specifically, we investigate how the uptake of credit from banks and other financial institutions affects the performance of non-farm household enterprises in Ghana. Section "Methodology and data" provides a more detailed discussion.

^6^Specifically, in this study access to finance is measured by the first principal component, based on a principal component analysis of the following three variables: (1) The percentage of new fixed investment financed by borrowing from local private commercial banks; (2) The percentage of new fixed investment financed by borrowing from foreign banks; (3) The percentage of new fixed investment financed by borrowing from loans from family or friends, money lenders, or other informal sources. Data of these variables are drawn from the Business Environment and Enterprise Performance Survey, which is jointly conducted by the World Bank and the European Bank for Reconstruction and Development. The survey covers information about firms from all countries of Central and Eastern Europe, the former Soviet Union, and Turkey.

^7^See World Bank (2015) for a more detailed discussion.

^8^In Ghana, the most commonly used criteria for defining the size of an enterprise is the number of employees. The Ghana Statistical Service (GSS) defines firms with less than 10 employees as micro-and-small enterprises.

^9^Source: http://www2.statsghana.gov.gh/nada/index.php/catalog/97/related_materials

^10^Another measure for firm productivity is total factor productivity (TFP), which has been adopted by, e.g., Gatti and Love (2008), Arnold et al. (2008), Chen and Guariglia (2013), Osei-Assibey (2013), and Bokpin et al. (2018). Specifically, the Cobb–Douglas production function, in the form of $Y_{i}=A_{i}{K_{i}}^{\beta_{1}}{L_{i}}^{\beta_{2}}$, is assumed, where a firm’s output ($Y_{i}$) depends on capital ($K_{i}$), labor ($L_{i}$), and total factor productivity ($A_{i}$), which is directly observable. To calculate $A_{i}$, we first take the natural logarithm of the production function and reach $\ln Y_{i}=\ln A_{i}+\beta_{1}\ln K_{i}+\beta_{2}\ln L_{i}\text{.}$ After rearrangement, $\ln A_{i}$ can be expressed as $\ln Y_{i}-\left(\beta_{1}\ln K_{i}+\beta_{2}\ln L_{i}\right)$, where the term $\left(\beta_{1}\ln K_{i}+\beta_{2}\ln L_{i}\right)$ can be obtained by estimating the following equation $\ln Y_{i}=\beta_{0}+\beta_{1}\ln K_{i}+\beta_{2}\ln L_{i}+\varepsilon_{i}$. Thus, $\ln A_{i}=\ln Y_{i}-\left(\beta_{1}\ln K_{i}+\beta_{2}\ln L_{i}\right)=\left(\beta_{0}+\varepsilon_{i}\right)$, and $TFP_{i}=A_{i}=e^{\left(\beta_{0}+\varepsilon_{i}\right)}\text{.}$ For instance, Osei-Assibey (2013, p.378) uses "the book value of such physical assets as machines, simple tools and equipment, land, vehicles, and etc." as the proxy for capital stock and "the total number of people engaged by the enterprise" as the indicator of labor input, and calculate TFP of non-farm household enterprises, based on the information from Ghana Living Standard Survey 2005/2006 (GLSS 5). However, our empirical analysis is using data from Ghana Living Standard Survey 2016/2017 (GLSS 7), from which information on the capital stock of non-farm household enterprises are missing. Therefore, we stick to labor productivity as the main variable of our interest. In our regression analyses, labor productivity is expressed in logarithm.

^11^Source: GLSS7, Section 10, Part A, Question 15.

^12^See, e.g., Garcia and Lensink (2019) for a more detailed discussion.

^13^For example, apprenticeship is an important form of professional trainings in Ghana. Usually, an apprentice learns the technology and business practices that are specific to the firm, in which the apprenticeship takes place. As the knowledge and skills acquired from the apprenticeship cannot be easily transferred and applied to other firms, most apprentices choose to establish their own enterprise after the apprenticeship, as self-employment allows them to replicate the knowledge and skills and reap the most benefits (Frazer, 2006).
