# Supplementary material for: Formal finance and household enterprise performance in Ghana: The gender dimension
Source: Front Psychol. 2022 Sep 30;13:887545. doi: 10.3389/fpsyg.2022.887545 (PMC9561877; doi:10.3389/fpsyg.2022.887545)
Supplement: Supplementary file 1 [file Data_Sheet_1.docx]

| **Appendix**  **Table A.1.** Descriptive Statistics: enterprises that applied for credit | | | | | |
| --- | --- | --- | --- | --- | --- |
| Variable | Obs | Mean | Std | Min | Max |
| **Dependent variable** |  |  |  |  |  |
| Labor productivity | 637 | 4.453 | 1.747 | -0.916 | 9.105 |
|  |  |  |  |  |  |
| **Explanatory variable** |  |  |  |  |  |
| Success | 732 | 0.807 | 0.395 | 0.000 | 1.000 |
| Fail | 732 | 0.193 | 0.395 | 0.000 | 1.000 |
|  |  |  |  |  |  |
| **Control variables** |  |  |  |  |  |
| *Head characteristics* |  |  |  |  |  |
| Age | 735 | 53.103 | 14.062 | 20.000 | 90.000 |
| Male | 735 | 0.616 | 0.487 | 0.000 | 1.000 |
| Female | 735 | 0.384 | 0.487 | 0.000 | 1.000 |
| Schooled | 735 | 0.810 | 0.393 | 0.000 | 1.000 |
| Not schooled | 735 | 0.190 | 0.393 | 0.000 | 1.000 |
| Married | 735 | 0.356 | 0.479 | 0.000 | 1.000 |
| Unmarried | 735 | 0.644 | 0.479 | 0.000 | 1.000 |
| *Enterprise characteristics* |  |  |  |  |  |
| Years of operation | 669 | 1.813 | 0.937 | 0.000 | 3.850 |
| Women labor | 735 | 0.687 | 0.446 | 0.000 | 1.000 |
| Household size | 735 | 3.850 | 2.990 | 1.000 | 15.000 |
| *Enterprise location* |  |  |  |  |  |
| Accra | 735 | 0.012 | 0.110 | 0.000 | 1.000 |
| Other urban | 735 | 0.503 | 0.500 | 0.000 | 1.000 |
| Rural coastal | 735 | 0.086 | 0.280 | 0.000 | 1.000 |
| Rural forest | 735 | 0.161 | 0.367 | 0.000 | 1.000 |
| Rural savannah | 735 | 0.238 | 0.426 | 0.000 | 1.000 |
| *Industry characteristics* |  |  |  |  |  |
| Manufacturing | 732 | 0.146 | 0.354 | 0.000 | 1.000 |
| Trade | 732 | 0.531 | 0.499 | 0.000 | 1.000 |
| Other services | 732 | 0.120 | 0.325 | 0.000 | 1.000 |
| Meals | 732 | 0.202 | 0.402 | 0.000 | 1.000 |

| **Table A.2.** Descriptive Statistics by gender: full sample | | | | | | | | | | | | |
| --- | --- | --- | --- | --- | --- | --- | --- | --- | --- | --- | --- | --- |
|  | **Male Group** | | | | | **Female Group** | | | | | **T-TEST** | |
|  | Obs | Mean | Std | Min | Max | Obs | Mean | Std | Min | Max | t-statistic | p-value |
| **Dependent variable** |  |  |  |  |  |  |  |  |  |  |  |  |
| Labor productivity (log) | 3875 | 4.240 | 1.288 | -0.799 | 10.645 | 2213 | 4.604 | 2.133 | -0.916 | 11.695 | 8.310*** | 0.000 |
|  |  |  |  |  |  |  |  |  |  |  |  |  |
| **Explanatory variable** |  |  |  |  |  |  |  |  |  |  |  |  |
| Not applied | 4362 | 0.896 | 0.305 | 0.000 | 1.000 | 2597 | 0.892 | 0.310 | 0.000 | 1.000 | -0.552 | 0.581 |
| Success | 4362 | 0.083 | 0.276 | 0.000 | 1.000 | 2597 | 0.088 | 0.283 | 0.000 | 1.000 | 0.662 | 0.508 |
| Fail | 4362 | 0.020 | 0.141 | 0.000 | 1.000 | 2597 | 0.020 | 0.140 | 0.000 | 1.000 | -0.109 | 0.913 |
|  |  |  |  |  |  |  |  |  |  |  |  |  |
| **Control variables** |  |  |  |  |  |  |  |  |  |  |  |  |
| *Entrepreneur characteristics* |  |  |  |  |  |  |  |  |  |  |  |  |
| age | 4363 | 55.127 | 13.840 | 17.000 | 95.000 | 2599 | 50.098 | 13.616 | 18.000 | 97.000 | -14.753*** | 0.000 |
| Schooled | 4363 | 0.807 | 0.395 | 0.000 | 1.000 | 2599 | 0.816 | 0.387 | 0.000 | 1.000 | 0.997 | 0.319 |
| Not schooled | 4363 | 0.193 | 0.395 | 0.000 | 1.000 | 2599 | 0.184 | 0.387 | 0.000 | 1.000 | -0.997 | 0.319 |
| Married | 4363 | 0.845 | 0.362 | 0.000 | 1.000 | 2599 | 0.316 | 0.465 | 0.000 | 1.000 | -52.847*** | 0.000 |
| Unmarried | 4363 | 0.155 | 0.362 | 0.000 | 1.000 | 2599 | 0.684 | 0.465 | 0.000 | 1.000 | 52.847*** | 0.000 |
| *Enterprise characteristics* |  |  |  |  |  |  |  |  |  |  |  |  |
| Years of operation | 4018 | 1.735 | 0.925 | 0.000 | 4.357 | 2390 | 1.745 | 0.937 | 0.000 | 4.595 | 0.657 | 0.445 |
| Women labor | 4362 | 0.710 | 0.435 | 0.000 | 1.000 | 2597 | 0.713 | 0.434 | 0.000 | 1.000 | 0.806 | 0.245 |
| Household size | 4363 | 4.612 | 3.393 | 1.000 | 18.000 | 2599 | 3.166 | 2.687 | 1.000 | 28.000 | -18.539*** | 0.000 |
| *Enterprise location* |  |  |  |  |  |  |  |  |  |  |  |  |
| Accra | 4363 | 0.012 | 0.109 | 0.000 | 1.000 | 2599 | 0.008 | 0.092 | 0.000 | 1.000 | -1.539 | 0.174 |
| Other urban | 4363 | 0.430 | 0.495 | 0.000 | 1.000 | 2599 | 0.600 | 0.490 | 0.000 | 1.000 | 13.970*** | 0.000 |
| Rural coastal | 4363 | 0.020 | 0.141 | 0.000 | 1.000 | 2599 | 0.152 | 0.359 | 0.000 | 1.000 | 21.517*** | 0.000 |
| Rural forest | 4363 | 0.217 | 0.412 | 0.000 | 1.000 | 2599 | 0.043 | 0.204 | 0.000 | 1.000 | -20.031*** | 0.000 |
| Rural savannah | 4363 | 0.321 | 0.467 | 0.000 | 1.000 | 2599 | 0.196 | 0.397 | 0.000 | 1.000 | -11.416*** | 0.000 |
| *Industrial characteristics* |  |  |  |  |  |  |  |  |  |  |  |  |
| Manufacturing | 4362 | 0.185 | 0.389 | 0.000 | 1.000 | 2597 | 0.193 | 0.394 | 0.000 | 1.000 | 0.729 | 0.466 |
| Trade | 4362 | 0.448 | 0.497 | 0.000 | 1.000 | 2597 | 0.449 | 0.498 | 0.000 | 1.000 | 0.151 | 0.880 |
| Other services | 4362 | 0.221 | 0.415 | 0.000 | 1.000 | 2597 | 0.219 | 0.413 | 0.000 | 1.000 | -0.267 | 0.789 |
| Meals | 4362 | 0.146 | 0.353 | 0.000 | 1.000 | 2597 | 0.139 | 0.346 | 0.000 | 1.000 | -0.712 | 0.476 |

| **Table A.3.** Descriptive Statistics by gender: enterprises that applied for credit | | | | | | | | | | | | |
| --- | --- | --- | --- | --- | --- | --- | --- | --- | --- | --- | --- | --- |
|  | **Male Group** | | | | | **Female Group** | | | | | **T-TEST** | |
|  | Obs | Mean | Std | Min | Max | Obs | Mean | Std | Min | Max | t-statistic | p-value |
| **Dependent variable** |  |  |  |  |  |  |  |  |  |  |  |  |
| Labor productivity (log) | 401 | 4.330 | 1.478 | 0.460 | 9.105 | 236 | 4.662 | 2.116 | -0.916 | 8.854 | 2.318** | 0.021 |
|  |  |  |  |  |  |  |  |  |  |  |  |  |
| **Explanatory variable** |  |  |  |  |  |  |  |  |  |  |  |  |
| Success | 452 | 0.803 | 0.398 | 0.000 | 1.000 | 280 | 0.814 | 0.390 | 0.000 | 1.000 | 0.373 | 0.710 |
| Fail | 452 | 0.197 | 0.398 | 0.000 | 1.000 | 280 | 0.186 | 0.390 | 0.000 | 1.000 | -0.373 | 0.710 |
|  |  |  |  |  |  |  |  |  |  |  |  |  |
| **Control variables** |  |  |  |  |  |  |  |  |  |  |  |  |
| *Entrepreneur characteristics* |  |  |  |  |  |  |  |  |  |  |  |  |
| age | 453 | 54.907 | 14.217 | 20.000 | 90.000 | 282 | 50.206 | 13.331 | 20.000 | 80.000 | -4.464*** | 0.000 |
| Schooled | 453 | 0.799 | 0.401 | 0.000 | 1.000 | 282 | 0.826 | 0.380 | 0.000 | 1.000 | 0.910 | 0.363 |
| Not schooled | 453 | 0.201 | 0.401 | 0.000 | 1.000 | 282 | 0.174 | 0.380 | 0.000 | 1.000 | -0.910 | 0.363 |
| Married | 453 | 0.148 | 0.355 | 0.000 | 1.000 | 282 | 0.691 | 0.463 | 0.000 | 1.000 | 17.918*** | 0.000 |
| Unmarried | 453 | 0.852 | 0.355 | 0.000 | 1.000 | 282 | 0.309 | 0.463 | 0.000 | 1.000 | -17.918*** | 0.000 |
| *Enterprise characteristics* |  |  |  |  |  |  |  |  |  |  |  |  |
| Years of operation | 412 | 1.812 | 0.923 | 0.000 | 3.850 | 257 | 1.815 | 0.962 | 0.000 | 3.807 | 0.657 | 0.445 |
| Women labor | 453 | 0.673 | 0.452 | 0.000 | 1.000 | 282 | 0.709 | 0.437 | 0.000 | 1.000 | 0.806 | 0.245 |
| Household size | 453 | 4.302 | 3.136 | 1.000 | 15.000 | 282 | 3.124 | 2.584 | 1.000 | 12.000 | -5.289*** | 0.000 |
| *Enterprise location* |  |  |  |  |  |  |  |  |  |  |  |  |
| Accra | 453 | 0.013 | 0.114 | 0.000 | 1.000 | 282 | 0.011 | 0.103 | 0.000 | 1.000 | -0.312 | 0.755 |
| Other urban | 453 | 0.457 | 0.499 | 0.000 | 1.000 | 282 | 0.578 | 0.495 | 0.000 | 1.000 | 3.210*** | 0.001 |
| Rural coastal | 453 | 0.024 | 0.154 | 0.000 | 1.000 | 282 | 0.184 | 0.388 | 0.000 | 1.000 | 7.839*** | 0.000 |
| Rural forest | 453 | 0.243 | 0.429 | 0.000 | 1.000 | 282 | 0.028 | 0.166 | 0.000 | 1.000 | -8.021*** | 0.000 |
| Rural savannah | 453 | 0.263 | 0.441 | 0.000 | 1.000 | 282 | 0.199 | 0.400 | 0.000 | 1.000 | -1.987** | 0.047 |
| *Industrial characteristics* |  |  |  |  |  |  |  |  |  |  |  |  |
| Manufacturing | 452 | 0.137 | 0.344 | 0.000 | 1.000 | 280 | 0.161 | 0.368 | 0.000 | 1.000 | 0.876 | 0.382 |
| Trade | 452 | 0.531 | 0.500 | 0.000 | 1.000 | 280 | 0.532 | 0.500 | 0.000 | 1.000 | 0.031 | 0.976 |
| Other services | 452 | 0.131 | 0.337 | 0.000 | 1.000 | 280 | 0.104 | 0.305 | 0.000 | 1.000 | -1.089 | 0.276 |
| Meals | 452 | 0.201 | 0.401 | 0.000 | 1.000 | 280 | 0.204 | 0.403 | 0.000 | 1.000 | 0.073 | 0.942 |

| **Table A.4.** Descriptive Statistics by gender: enterprises that applied for credit and succeeded | | | | | | | | | | | | |
| --- | --- | --- | --- | --- | --- | --- | --- | --- | --- | --- | --- | --- |
|  | **Male Group** | | | | | **Female Group** | | | | | **T-TEST** | |
|  | Obs | Mean | Std | Min | Max | Obs | Mean | Std | Min | Max | t-statistic | p-value |
| **Dependent variable** |  |  |  |  |  |  |  |  |  |  |  |  |
| Labor productivity (log) | 322 | 4.424 | 1.443 | 0.693 | 9.105 | 190 | 4.672 | 2.168 | -0.916 | 8.854 | 1.552 | 0.123 |
|  |  |  |  |  |  |  |  |  |  |  |  |  |
| **Explanatory variable** |  |  |  |  |  |  |  |  |  |  |  |  |
| Success | 363 | 1.000 | 0.000 | 1.000 | 1.000 | 228 | 1.000 | 0.000 | 1.000 | 1.000 |  |  |
|  |  |  |  |  |  |  |  |  | a |  |  |  |
| **Control variables** |  |  |  |  |  |  |  |  |  |  |  |  |
| *Entrepreneur characteristics* |  |  |  |  |  |  |  |  |  |  |  |  |
| age | 363 | 54.642 | 14.109 | 20.000 | 90.000 | 228 | 49.877 | 13.222 | 20.000 | 80.000 | -4.094*** | 0.000 |
| Schooled | 363 | 0.799 | 0.401 | 0.000 | 1.000 | 228 | 0.816 | 0.389 | 0.000 | 1.000 | 0.504 | 0.614 |
| Not schooled | 363 | 0.201 | 0.401 | 0.000 | 1.000 | 228 | 0.184 | 0.389 | 0.000 | 1.000 | -0.504 | 0.614 |
| Married | 363 | 0.149 | 0.356 | 0.000 | 1.000 | 228 | 0.711 | 0.455 | 0.000 | 1.000 | 16.743*** | 0.000 |
| Unmarried | 363 | 0.851 | 0.356 | 0.000 | 1.000 | 228 | 0.289 | 0.455 | 0.000 | 1.000 | -16.743*** | 0.000 |
| *Enterprise characteristics* |  |  |  |  |  |  |  |  |  |  |  |  |
| Years of operation | 330 | 1.835 | 0.933 | 0.000 | 3.850 | 208 | 1.783 | 0.950 | 0.000 | 3.807 | -0.630 | 0.529 |
| Women labor | 363 | 0.675 | 0.454 | 0.000 | 1.000 | 228 | 0.722 | 0.431 | 0.000 | 1.000 | 1.247 | 0.213 |
| Household size | 363 | 4.512 | 3.220 | 1.000 | 15.000 | 228 | 3.096 | 2.550 | 1.000 | 12.000 | -5.624*** | 0.000 |
| *Enterprise location* |  |  |  |  |  |  |  |  |  |  |  |  |
| Accra | 363 | 0.017 | 0.128 | 0.000 | 1.000 | 228 | 0.004 | 0.066 | 0.000 | 1.000 | -0.312 | 0.755 |
| Other urban | 363 | 0.452 | 0.498 | 0.000 | 1.000 | 228 | 0.579 | 0.495 | 0.000 | 1.000 | 3.028*** | 0.003 |
| Rural coastal | 363 | 0.019 | 0.138 | 0.000 | 1.000 | 228 | 0.180 | 0.385 | 0.000 | 1.000 | 7.246*** | 0.000 |
| Rural forest | 363 | 0.231 | 0.422 | 0.000 | 1.000 | 228 | 0.035 | 0.184 | 0.000 | 1.000 | -6.632*** | 0.000 |
| Rural savannah | 363 | 0.281 | 0.450 | 0.000 | 1.000 | 228 | 0.202 | 0.402 | 0.000 | 1.000 | -2.169** | 0.031 |
| *Industrial characteristics* |  |  |  |  |  |  |  |  |  |  |  |  |
| Manufacturing | 363 | 0.124 | 0.330 | 0.000 | 1.000 | 228 | 0.154 | 0.361 | 0.000 | 1.000 | 1.021 | 0.308 |
| Trade | 363 | 0.554 | 0.498 | 0.000 | 1.000 | 228 | 0.539 | 0.500 | 0.000 | 1.000 | -0.338 | 0.735 |
| Other services | 363 | 0.105 | 0.307 | 0.000 | 1.000 | 228 | 0.096 | 0.296 | 0.000 | 1.000 | -0.321 | 0.749 |
| Meals | 363 | 0.218 | 0.413 | 0.000 | 1.000 | 228 | 0.211 | 0.409 | 0.000 | 1.000 | -0.204 | 0.838 |
